# Biodegradable Silk Fibroin Nanocarriers to Modulate Hypoxia Tumor Microenvironment Favoring Enhanced Chemotherapy

**DOI:** 10.3389/fbioe.2022.960501

**Published:** 2022-07-22

**Authors:** Li Bin, Yuxiao Yang, Feiyu Wang, Rong Wang, Hongxin Fei, Siliang Duan, Linling Huang, Na Liao, Shimei Zhao, Xinbo Ma

**Affiliations:** ^1^ Department of Biochemistry and Molecular Biology, Medical College, Guangxi University of Science and Technology, Liuzhou, China; ^2^ Second Clinical Medical College, Medical College, Guangxi University of Science and Technology, Liuzhou, China

**Keywords:** biocompatibility, hypoxia, chemotherapy, atovaquone, breast cancer

## Abstract

Biopolymer silk fibroin (SF) is a great candidate for drug carriers characterized by its tunable biodegradability, and excellent biocompatibility properties. Recently, we have constructed SF-based nano-enabled drug delivery carriers, in which doxorubicin (Dox) and atovaquone (Ato) were encapsulated with Arg-Gly-Asp-SF-Polylactic Acid (RSA) to form micellar-like nanoparticles (RSA-Dox-Ato NPs). The RGD peptide was decorated on micellar-like nanoparticles, promoting tumor accumulation of the drug. Meanwhile, Ato, as a mitochondrial complex III inhibitor inhibiting mitochondrial respiration, would reverse the hypoxia microenvironment and enhance chemotherapy in the tumor. *In vitro*, the biopolymer alone showed extremely low cytotoxicity to 4T1 cell lines, while the RSA-Dox-Ato demonstrated a higher inhibition rate than other groups. Most significantly, the ROS levels in cells were obviously improved after being treated with RSA-Dox-Ato, indicating that the hypoxic microenvironment was alleviated. Eventually, SF-based targeted drug carrier provides biocompatibility to reverse hypoxia microenvironment *in vivo* for enhancing chemotherapy, strikingly suppressing tumor development, and thereby suggesting a promising candidate for drug delivery system.

## Introduction

While the 5-year survival outcome in traditional antitumor modalities has clinical progress, the mortality in patients with advanced tumors remained undiminished owing to metastasis and recurrence (Zhang et al., 2016; Zhang et al., 2017; Zhang et al., 2018a; Zhang et al., 2020a; Cai et al., 2022). Hypoxic tumor microenvironment (TME) is gradually recognized as an essential factor in tumor progression, especially in multidrug resistance, radiosensitivity, immunologic escape, and metastasis (Jiang et al., 2020; Jin et al., 2021). According to previously described, the lower oxygen levels in locally advanced tumors (less than 2.5 mm Hg) compared with that in normal tissue (20–150 mm Hg) (Vaupel et al., 2007). Obviously, the difference is mainly caused by high oxygen consumption for tumor proliferation and abnormal angiogenesis in the hypoxic tumor, resulting imbalance in oxygen supply and depletion (Ye et al., 2018). Against this background, probing a robust TME-modulating nanocarrier as preludes for tumor combination therapies is extremely crucial.

To reverse the oxygen imbalance, a series of therapeutic strategies have been proposed around the hypoxic tumor microenvironment for improving chemotherapy efficiency. For instance, oxygen-carrying and oxygen-generating with multifunctional nanodrug delivery systems were performed to overcome tumor hypoxia (Zhang et al., 2018b; Zhao et al., 2018; Zuo et al., 2018; Zhang et al., 2020b; Huang et al., 2020; Zhao et al., 2020). Such strategies may still have restricted therapeutic effects, especially toward unlimited proliferation of tumor cells with rapid oxygen consumption (Li et al., 2018; Wang et al., 2019; Li et al., 2020; Xia et al., 2020). Recently, the mitochondria-targeted metabolism strategy may be a promising treatment to inhibit proliferation and hypoxia in tumor. The mechanism of mitochondria-targeted strategy is inhibition of mitochondrial complex III in Krebs (TCA) cycle which deplete the oxygen to produce ATP for energy metabolism and the cell proliferation (Jeena et al., 2019; Frattaruolo et al., 2020). To date, atovaquone (Ato), known as an FDA-approved antimicrobial agent, competitively replaced the ubiquinol to occupy the active center of mitochondrial complex III, interrupting the respiratory chain in breast cancer cells (Xia et al., 2019; Cheng et al., 2020). In addition, mitochondria-targeted atovaquone increases tumor-infiltrating CD4^+^ T cells in PD-1 blockade immunotherapy (Huang et al., 2022). However, circulation time, targeted effect, and hydrophilia featured in small molecular drugs have limited the clinical antitumor and therapeutic effect.

Silk fibroin (SF), a promising biomaterial that has attracted substantial attention, is a natural biopolymeric protein with good biocompatibility and drug-loading capability (Zhao et al., 2015; Hu et al., 2018; Mao et al., 2018). Under low pH conditions, doxorubicin-loaded silk nanoparticles readily release the payloads due to changes in surface properties (Seib et al., 2013; Xia et al., 2014; Montalbán et al., 2018). What’s more, SF as a protein nanoparticle could be decomposed in the lysosome and rapidly eliminated by kidneys (Nguyen et al., 2019; Li et al., 2021). Compared with inorganic nano-delivery systems, the biodegradable SF nanocarriers have no long-term toxicity problems *in vivo* applications (Mathur and Gupta, 2010; Subia et al., 2015). In addition, TME-responsive SF-based nanocarriers with outcoming biocompatibility enable efficient therapeutics to realize the lysosomes targeting and escape (Tan et al., 2019; Zhang et al., 2020c; Yu et al., 2022). Considering off-target toxicity in normal tissues, nanocarrier was modified by an active targeting agent (RGD) to enhance the injected doses of drugs end up in tumor tissue. According to previously reports, RGD is a targeted small molecule peptide that specifically interacts with integrin ɑvβ3 overexpressed on tumor cells (Meyer et al., 2006; Subia et al., 2014; Mao et al., 2018; Bari et al., 2021). The surprising case of RGD decorated polymer-based nano-micelles encouraged us to investigate whether the SF-based nanocarriers decorated with RGD as multifunctional nanoplatforms enables low side-effect and efficacy in chemotherapy treatment.

**SCHEME 1:**
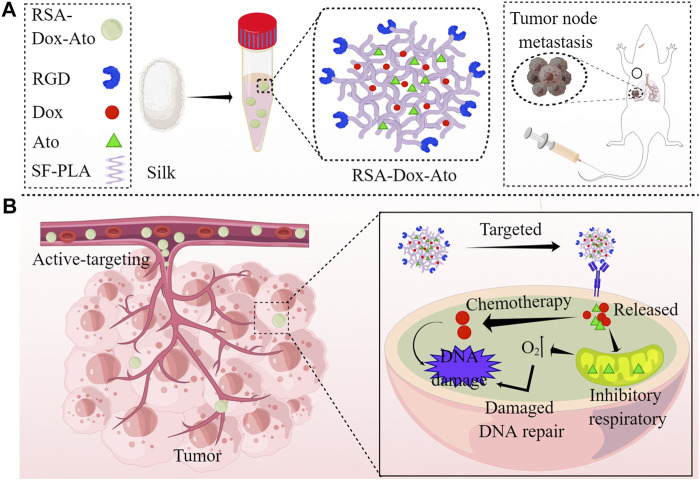
Schematics demonstration the RSA-Dox-Ato alleviating tumor hypoxia and improving the antitumor efficacy (by Figdraw). **(A)** Scheme illustrating the synthesis of RSA-Dox-Ato NPs; **(B)** The mechanism of RSA-Dox-Ato NPs alleviate tumor hypoxia *in vivo*.

Herein, we therefore custom-constructed SF-based biocompatible nanoplatform as a nano-enabled drug delivery system for targeting the tumor in a highly-efficient manner, realizing TME-responsive drug release to enhance chemotherapy by reversing hypoxia in the tumor (as shown in Scheme 1A). For this system, SF proteins were FDA-approved natural materials in medical applications with outstanding biodegradability under weakly acidic environments and proteases. Therefore, payloads of silk nanoparticles would be dissociated to release Dox for enhanced chemotherapy upon encountering a lower pH environment. Meanwhile, the released atovaquone competitively banded to mitochondrial complex III, obstructing mitochondrial respiration and oxygen consumption to modulate hypoxia in the tumor (as shown in Scheme 1B). Therefore, this work focuses on the reversal of hypoxic tumor microenvironment by promising SF-based nano-systems to improve the chemotherapy efficacy, providing a new angle in fighting tumors.

## Materials and Methods

### Materials

All reagents in this study were purchased from commercial suppliers without further purification. The paraformaldehyde (4%), sodium chloride (NaCl), disodium hydrogen phosphate (Na_2_HPO_4_), sodium dihydrogen phosphate (NaH_2_PO_4_), potassium chloride (KCl), potassium hydroxide (KOH) were purchased from Sigma-Aldrich (Shanghai, China). RPMI-1640 incomplete medium with 1% (v/v) penicillin-streptomycin, trypsin-EDTA digestion supplemented with 0.25% trypsin and 0.02% EDTA were purchased from KeyGen Biotech Co., Ltd., (Nanjing, China). The reactive oxygen level in tumor cells was observed by CM-H2DCFDA reagent kit under laser scanning confocal microscope. About 10% (v/v) serum (FBS) was supplemented in cell culture media (RPMI-1640). Dimethyl sulfoxide (DMSO) and thiazolyl blue (MTT) were obtained from Solarbio Technology Ltd (Beijing, China). Doxorubicin (DOX) was obtained from Titan technology Co. Ltd (Shanghai, China). The mitochondrial respiratory chain was competitively inhibited by atovaquone (Marck KGaA, Shanghai, China). Silk protein (SF) and SF-polylactic acid (SF-PLA) was bought from Hongxin Liyu Fine Chemical Ltd (Wuhan, Hubei, China). RGD peptide (The purity >90%) was purchased from PeptideValley (Nanjing, China). Hoechst 33,342 was obtained from Beyotime Biotechnology Ltd (Shanghai, China). The mouse-derived 4T1 cancer cells were incubated in RPMI-1640 complete media containing with 10% FBS, and obtained from Shanghai Institute of Cells (Shanghai, China). All-female BLAB/c mice (SPF, 5-weeks-old, weighing 16–19 g, Animal Certificate NO: SCXK (Beijing) 2019–0010) were purchased from SPF Biotechnology Co., Ltd., (Beijing, China), and housed under room temperature with the pathogen-free environment. All mice experiments were approved by the Institutional Animal Ethics Committees of Guangxi University of Science and Technology (Liuzhou, China). All experiments in this study were performed with deionized water (Persee, 18.2 MΩ cm-1).

### Preparation of SF-Based Nanocarriers

SF-based nanocarriers were synthesized by the oil-in-water emulsion solvent diffusion method as described in previously reported (Qiao et al., 2015; Mao et al., 2018; Wei et al., 2021). Firstly, the EDC and NHS were successively added with RGD solution to activate the carboxyl group. The NH_2_-SF-PLA (SA) was added to synthesize the RSA under pH 5.0 at room temperature. RSA was collected after freeze-drying and stored at 4°C. Then, the fabrication process includes dissolution, filtration, agitation, centrifugation, sonication, and lyophilization. For instance, 2.0 mg of doxorubicin (Dox) and 1.9 mg of atovaquone (Ato) were firstly dissolved using 5 ml of chloroform. The dissolved solution was then dropped into 25 ml of SF-based micelles (the concentration of SF-based micelles was 0.5 mg/ml in deionized water) at the speed of six drops/minute. Ultrasound continued for 25 min after the solution was added. The mixture was then dialyzed in deionized water. After lyophilization, the SA-Dox-Ato and RSA-Dox-Ato nano micelles were collected and saved at 4°C.

### Characterization

To observe the RSA-Dox-Ato NPs, the morphology was visualized with a transmission electron microscope (HC-1, Hitachi, Japan). The UV-vis spectrophotometer UV-2600 (Shimadzu, Japan) was successfully performed to record the absorption spectra of Ato, Dox, RSA, and RSA-Dox-Ato. To investigate the release behavior under different pH environment, the RSA-Dox-Ato NPs were resuspended in PBS (1 ml) with pH 5.0 and pH 7.4, respectively. Then, the sediment of RSA-Dox-Ato NPs was collected at a fixed time after centrifugation, while the precipitate was redispersed with PBS under different pH conditions. Then, the absorbance of Ato and free Dox were respectively monitored by UV-vis spectrophotometer UV-2600 (SHIMADZU, Japan) at room temperature.

### Cell Culture

The mouse breast cancer cells (4T1) were cultured for cytotoxicity studies *in vitro*. First, the 4T1 cells were cultured with a complete medium (prepared with 100 U/ml penicillin, 10% (v/v) fetal bovine serum (FBS), 100 μg/ml streptomycin, and RPMI-1640 incomplete medium) in a cell incubator (BPN-80CH, Blue Pard, Shanghai) at 37°C with 5% CO_2_ under a humidified atmosphere. When the 4T1 cells reached 80% confluence, discarded the medium and washed all dishes with PBS (pH 7.4) three times. Then, the cells were digested with trypsin-EDTA under 37°C and collected in 15 ml sterile centrifugal tubes for subculturing in a fresh RPMI-1640 medium.

### Cytotoxicity Assay

The MTT assay was employed to investigate the cytotoxicity of RSA-Dox-Ato for 4T1 cells *in vitro*. Firstly, 4T1 cells were seeded under standard protocol and subcultured in a multi-well plate with 100 μL of RPMI-1640 medium at a density of 5 × 10^3^ cells/well. After 12 h, all groups were respectively treated with PBS, Dox, RSA-Dox, and RSA-DOX-Ato at different concentrations (*n* = 3) as an experimental procedure. Then, the time-dependence effect of cell viability was respectively evaluated within 12 and 24 h. Subsequently, the medium of all groups was removed and washed three times with PBS for 5 min, re-incubated with MTT solution (100 μL) at a concentration of 5 mg/ml for 4 h according to cytotoxicity protocol. All these processed 4T1 cells were incubated under a humidified environment at 37°C with 5% CO_2_. Meanwhile, 150 μL of DMSO was applied to dissolve the solid crystals following washed by PBS (pH 7.4) in each well. After that, the absorbance of samples in each well was reported using a microplate reader at 490 nm (Victor Nivo 3S, US). The cytotoxicity (%) of nanodrugs was evaluated and calculated as OD treatment/OD control × 100%.

### Evaluation of Endocytosis

The receptor-mediated endocytosis in 4T1 cells of RSA-Dox-Ato NPs was assessed utilizing confocal laser scanning microscopy (CLSM, Nikon). The laser confocal dish was seeded with 5 × 10^4^ cells per well and placed in a cell incubator (37°C, 5% CO_2_) for 24 h. These tumor cells were seeded with RPMI-1640 medium contained 100 U/ml penicillin, 10% (v/v) serum (FBS), 100 μg/ml streptomycin. After 24 h incubation, RSA-Dox-Ato (500 μL, 100 μg/ml) and Dox (500 μL, 100 μg/ml) were respectively added and co-cultured with 4T1 cells at 37°C with 5% CO_2_. Later, the supernatant of each dish was removed, and the 4T1 cells in each dish were washed three times with PBS. Subsequently, 4% paraformaldehyde (1 ml) in PBS (pH 7.4) was added to dishes to fixed tumor cells for 20 min. Then, triton X-100 (1 ml, 0.1% v/v) was employed to wash cells three times. Afterward, the cell nucleus was stained using Hoechst 33,342 (blue) in the dark field for 20 min. Finally, the cells were washed with PBS (pH 7.4) three times, and the fluorescence images were obtained by confocal microscopy.

### The Oxygen Consumption Rate

The oxygen consumption rate (OCR) was detected as the previous methods (Diepart et al., 2012; Xia et al., 2019; Xia et al., 2020). Briefly, the 4T1 cells were seeded in a 12-well plate within 24 h at a cell concentration of about 3 × 104 cells/well. All cells in the 12-well plate were incubated with RPMI-1640 medium containing 100 U/ml penicillin, 10% (v/v) fetal bovine serum (FBS), and 100 μg/ml streptomycin. To test the oxygen concentration in the medium, all groups treated with nanodrugs were placed in the anaerobic environment with AnaeroPack system. Then, the oxygen concentration in the medium was continuously evaluated for 4 h at a multi-time-point using a dissolved oxygen instrument (Huihe intelligent Electronic Instrument Co., Ltd., China). The OCR level (%) was calculated after oxygen concentration normalized.

### ROS Detection *In Vitro*

Inspired by the oxygen consumption under medium, the ROS in 4T1 cells treated with reactive oxygen species detection assay kit (CM-H2DCFDA) was furtherly observed under fluorescence microscopy. All operations follow the protocol. Rosup as a ROS activator was used to induce ROS production. Firstly, 4T1 cells with a density of 5 × 10^4^ cells/well were incubated in a 12-well plate under complete RPMI 1640 medium at 37°C with 5% CO_2_. DCFH-DA After an incubation of 24 h, all groups were respectively treated with RSA-Dox (500 μL, Dox concentration is 12.5 μg/ml) + Rosup (50 μg/ml) and RSA-Dox-Ato (500 μL, Dox concentration is 12.5 μg/ml) + Rosup (50 μg/ml) for 2 h. The control group was added with PBS in plate. The H2DCFDA was diluted in RPMI 1640 medium without serum and injected into chambers of the 12-well plate. After incubated for 15 min in a cell incubator (37°C, 5% CO_2_), the culture media was respectively removed, and 4T1 cells were further added with PBS (pH 7.4) to wash the H2DCFDA. Then, the cells were co-stained with Hoechst 33,342 in the dark field for 15 min. Subsequently, 4T1 cells were fixed with 4% paraformaldehyde (1 ml) in PBS (pH 7.4) for 20 min. Finally, the cells were washed three times with PBS and observed with an inverted fluorescence microscope (IX73, OLYMPUS). The generated fluorescence was visualized under 495/529 nm and analyzed by ImageJ software.

### Scratch Assay

In order to assess the influence of RSA-Dox-Ato in cell migration, the scratch assay as a direct and economical approach was adopted *in vitro*. In short, the cells were incubated into each well of a 6-well plate and co-incubated with RPMI 1640 medium with 100 U/ml penicillin, 10% FBS containing, and 100 μg/ml streptomycin. Afterward, the sterile pipette was used to wound the monolayer 4T1 cells, and the cell debris was removed by washing three times with PBS. Subsequently, all treatment groups were incubated with Dox (1 ml, Dox concentration is 5 μg/ml), RSA-Dox (1 ml, Dox concentration is 5 μg/ml) and RSA-Dox-Ato (1 ml, Dox concentration is 5 μg/ml), while the control group was injected with 1 ml of PBS (pH 7.4). After incubation for 24 h, the images of 4T1 cells were observed using an inverted microscope and analyzed by ImageJ software.

### The 4T1 Tumor-Bearing Mice Models

About 5 weeks old healthy female Balb/c mice (About 20 g/mouse) were obtained and housed under pathogen-free conditions. After a week, the 4T1 tumor-bearing mice models were built by subcutaneous injection with 4T1 cells (5 × 10^6^ cells/mouse, 100 μL) into the right forelimb. Then, the tumor volume on mouse was monitored and measured by a vernier caliper after 6 days. Meantime, the tumor volumes were calculated as length × width2 × 1/2. Then, the 4T1 tumor-bearing mice models were divided randomly after the tumor volume reached about 100 mm^3^.

### Antitumor Efficacy *In Vivo*

The 4T1 tumor-bearing mice models were divided randomly into the following five groups, including the PBS group, RSA group, Dox group, RSA-Dox, and RSA-Dox-Ato. The treatment groups were injected intravenously with RSA, Dox, RSA-Dox, and RSA-Dox-Ato, while the PBS group as control was treated with PBS every other day. Meanwhile, the tumor-volume change and mouse weight were recorded to assess the therapeutic effects. Finally, all treated mice were weighted and humanely sacrificed, and tumors were collected to assess the inhibition effect. In addition, the major organs were isolated and saved in 4% paraformaldehyde for further histopathology experiments.

### Inhibition of Metastasis

To further investigate tumor metastasis, the xenograft-bearing mice were treated with nanodrugs *in vivo*. The lungs in mice were collected and washed three times in PBS. The metastasis nodules in the lung were counted and calculated to analyze the suppression efficiency of nanodrugs. Afterward, the lungs were obtained from mice and fixed in 4% (w/v) paraformaldehyde solution for metastasis evaluation in histopathology.

### Histopathology Analysis

The major organs (heart, liver, spleen, lung, and kidney) and tumors from the treated mice in the antitumor experiment were collected and were fixed in 4% paraformaldehyde solution, followed by paraffin embedding. After that, the tumors and the major organs were co-stained for immunohistochemical analyses. All of the sections from mice were collected and imaged by an inverted microscope for histopathology analysis.

### Statistical Analysis

All statistical analyses were conducted with a t-test in Excel with no outliers (two-sided tests). All data in pictures were presented as means ± standard deviation (n > 2, *p < 0.05, which represented the significant differences).

## Results

### Characterization of the RSA-Dox-Ato Nano-Micelles

**FIGURE 1:**
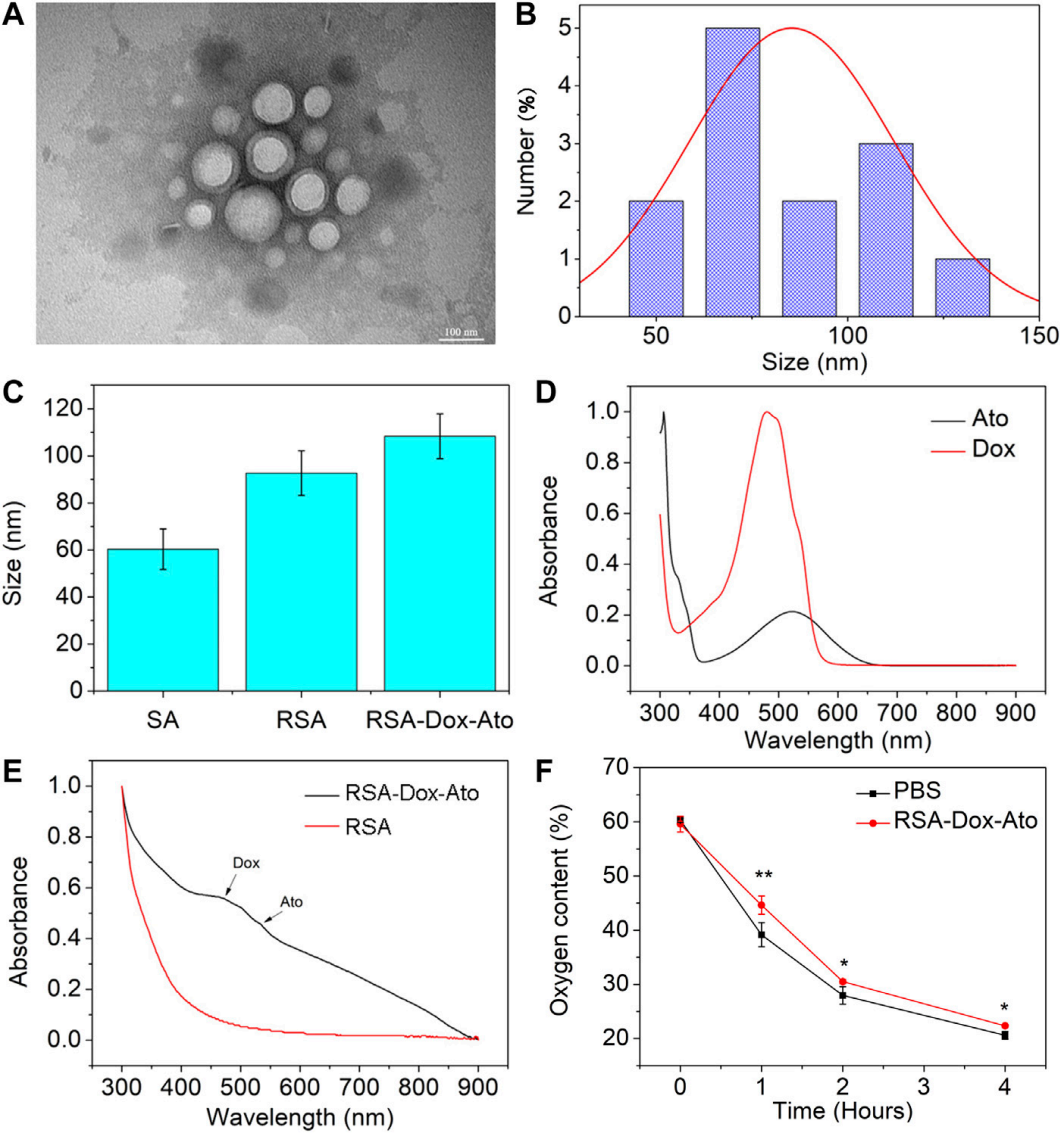
Characterization of SF-based nanoparticles. **(A)** TEM images of the RSA-Dox-Ato NPs. **(B)** The size distribution of the RSA-Dox-Ato NPs; **(C)** The mean hydrodynamic diameter distribution of RSA NPs (92.67 ± 9.50 nm) and RSA-Dox-Ato NPs (114.67 ± 14.29 nm); **(D)** UV-vis spectra of Ato and Dox; **(E)** UV-vis spectra of RSA-Dox-Ato NPs and SA NPs; **(F)** Oxygen concentration in the cell medium after treatment with and without Ato under an anaerobic environment.

According to the previous reports, SF-based nano-carries were fabricated utilizing the oil-in-water emulsion solvent diffusion method (Qiao et al., 2015; Mao et al., 2018; Wei et al., 2021). The Dox and Ato were firstly enveloped with RGD-SF-PLA (RSA) amphiphilic polymers (as shown in Scheme 1). Then, the synthesized RSA-Dox-Ato nano-micelles were furtherly characterized using a TEM micrograph. The nanoparticles of RSA-Dox-Ato had a uniformly dispersed spherical morphology, as indicated in the TEM micrograph illustrated in Figure 1A. The mean diameters were 85.38 ± 26.75 nm, as displayed in Figure 1B, indicating an excellent blood circulation time for Dox and Ato *in vivo*. In Figure 1C, the mean hydrodynamic diameter of RSA NPs was 92.67 ± 9.50 nm, which was investigated utilizing dynamic light scattering. For RSA-Dox-Ato NPs, the mean hydrodynamic diameter was detected to be 114.67 ± 14.29 nm, which was higher than that of RSA NPs. The mean hydrodynamic diameter of RSA-Dox-Ato NPs had no obvious changes for 6 days, preliminary indicating the stability under room temperature (as shown in Supplementary Figure S1). The zeta potential of the RSA NPs was -24.73 ± 1.98 mV (as shown in Supplementary Figure S2). To further evaluate the RSA-Dox-Ato NPs, the absorption spectra of Dox and Ato presented, respectively, strong unimodal pink at 480 and 524 nm, as depicted in Figure 1D. After synthesizing nanomicelles, two absorption peaks were observed with the RSA-Dox-Ato NPs, which correspond to the Dox and Ato spectrograms, as shown in Figure 1E. According to the pioneering reports, the silk protein-based nanocarriers were observed with excellent drug release behavior under an acidic environment. Notably, the acidic microenvironment appeared in the most tumors due to alter tumor vascularization and insufficient oxygen supply for glucose metabolism. Triggered and controlled SF-based nanomicelles release system was an essential factor for nanomedicine. Therefore, the release behavior of SF-based nanoparticles was evaluated under different pH conditions. As illustrated in Supplementary Figure S3, the release ratio of Dox under an acidic environment was higher than that in a slightly alkaline environment (pH 7.4), evidencing a pH-dependent release behavior. As the Ato serves as a mitochondrial inhibitor, we envisioned that hypoxia in the tumor environment could be alleviated to enhance the chemotherapy. To this end, the oxygen consumption rate (OCR) in the medium was monitored utilizing a dissolved oxygen instrument under an anaerobic environment with AnaeroPack system. As shown in Figure 1F, the treatment group with Ato revealed a higher mean dissolved oxygen concentration in medium (44.63 ± 1.69%) than the PBS group (39.17 ± 2.23%) within 1 h, indicating inhibition of the oxygen consumption in mitochondrial could be conducive to hypoxia alleviation.

### Cytotoxicity *In Vitro*

**FIGURE 2:**
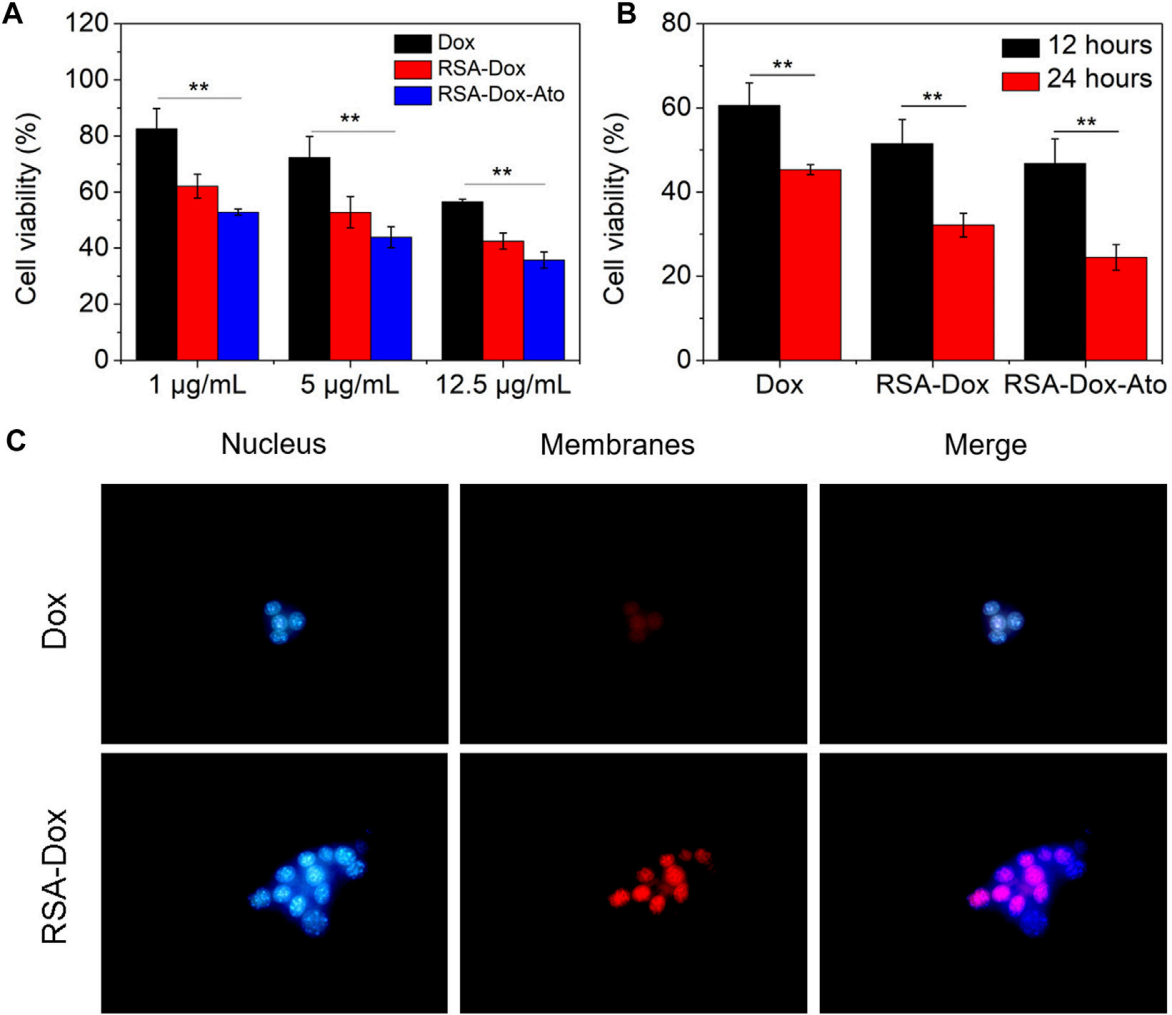
The cell cytotoxicity and endocytosis were evaluated *in vitro*. **(A)** The cell cytotoxicity of 4T1 cells in a dose-dependent manner was recorded by MTT assay *in vitro* (Dox, RSA-Dox, RSA-Dox-Ato). **(B)** The cell cytotoxicity of 4T1 cells in a time-dependent manner was recorded by MTT assay *in vitro* (Dox, RSA-Dox, RSA-Dox-Ato). **(C)** The endocytosis of 4T1 cells was observed by microscope with a ×40 objective *in vitro*. (*n* = 5, ∗*p* < 0.05, ∗∗*p* < 0.01).

To evaluate the cytotoxicity of Dox and Ato released by the prepared nanoparticles on 4T1 cells. The SF drug-loaded nanoparticles were incubated with 4T1 cancer cells for 12 h or 24 h with different concentrations, and cell viability was measured by MTT assay. As indicated in Figure 2A and Figure 2B, the cell viability of 4T1 cells was gradually decreased after treatment with nanodrugs under 37°C (5% CO_2_), indicating a dose-dependent relationship. Figure 2A shows that when cells were treated with a concentration drug of 1 μg/ml for 12 h, the cell survival rate was 82.6% in the Dox group, 62.1% in the RSA-Dox group, and 59.9% in the RSA-Dox-Ato group, and there was a significant difference. The results revealed that SF-based nanocarriers with RGD could enhance drug concentration in cells, and Ato could contribute to improved toxicity. What’s more, the cell viability demonstrated a time-dependent manner after treatment with nanodrugs at different times. As shown in Figure 2B, groups treated respectively with varying drugs for 24 h, including Dox, RSA-Dox, and RSA-Dox-Ato, displayed a higher antitumor effect than that in 12 h. Meanwhiles, the survival rate of 4T1 cells with high concentration drug treatment within 24 h, could be seen that the RSA-Dox group had a higher inhibition rate than the Dox group at the same concentration, regardless of whether the drug was treated within 12 h or 24 h. The increased inhibition efficacy of RSA-Dox was observed due to the RGD plays a better targeting ability to enable Dox enrichment in cells. It was worth noting that the RSA-Dox-Ato group exhibited the lowest cell viability than other treatment groups, even at a low concentration of 1 μg/ml, implying the outstanding antitumor efficacy of SF-based nanocarriers.

### The Evaluation of Endocytosis

Considering the internalization as a major biological barrier, the targeting strategy of RGD modified with SF-based nanocarriers was furtherly evaluated to improve endocytosis. To confirm the hypothesis, 4T1 cells were firstly treated with RSA-Dox and free Dox, then stained with Hoechst 33,342 and photographed. As depicted in Figure 2C, the RSA-Dox NPs group had stronger fluorescence than those in the DOX group, demonstrating that RGD modified SF-based nanocarrier contributed to Dox internalization by 4T1 cells. Therefore, SF-based drug-loaded nanoparticles modified with RGD enabled drugs to specifically bind with tumor cells, realizing targeted drug aggregation in tumors and off-target toxicity reduction.

### The Wound Healing Assays

**FIGURE 3:**
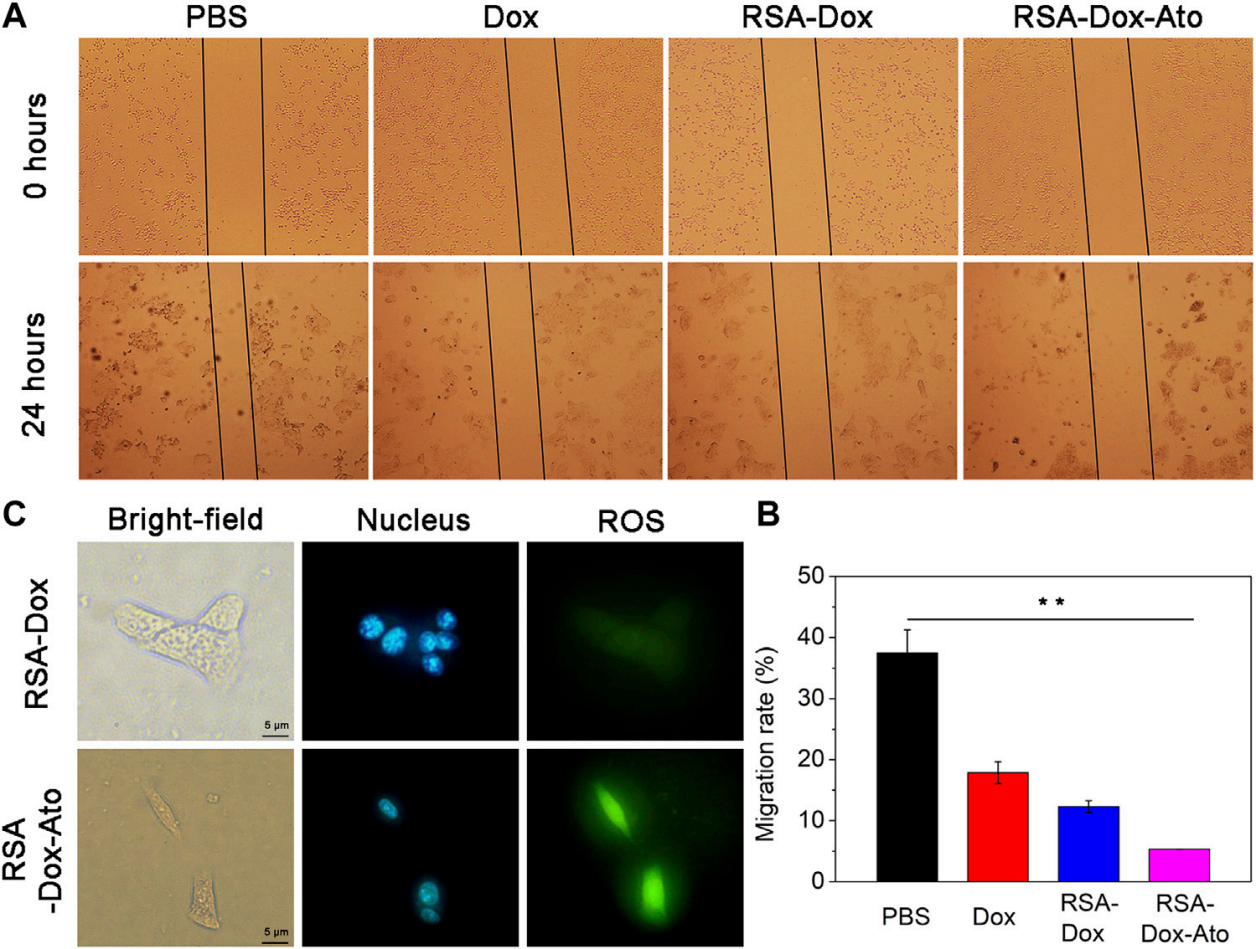
The effects of SF-based nanomicelles on cell migration and hypoxia. **(A)** The effects of drugs on cell migration in 4T1 cells after treatment within 0 hour and 24 hours (all cells were observed by microscope with a 4× objective); **(B)** The migration rate was calculated after various therapies; **(C)** The images of SF-based NPs against 4T1 cells hypoxia after various treatments.

Given the inhibition of nanodrugs loaded-Dox for tumor cells in previous reporters, the scratch assays, as an economical and easy processing method, were carried out to evaluate further the cell migration. In this study, the healing of scratch in cell plates by treating SF-based nanocarriers with the same concentration of Dox was observed and imaged. As shown in Figures 3A,B, the wound area of the PBS group without nano-drug treatment was narrowest, indicating that 4T1 cells had the fastest wound healing within 24 h. While wound healing of the 4T1 cells treated with Dox and silk fibroin nanodrugs was slower. It was worth noting that 4T1 cells were treated with RSA-Dox-Ato, resulting the extremely slow speed of wound healing after 24 h. That was to say, the RSA nanocarriers were capable of inhibiting tumor migration *in vitro*.

### Intracellular ROS Detection

ROS levels with appropriate improvement often favor cell proliferation, whereas an excessive number of ROS inhibit cell viability. Tumor cells lack oxygen owing to proliferation too fast, leading to large amounts of ROS intracellular production, so it is prone to trigger oxidative stress response leading to cell damage and apoptosis (Mirzaei et al., 2021). Recently, it has been reported that Ato alleviated tumor hypoxia by intervening in the function of mitochondrial complex III and increasing the level of intracellular ROS (Zhao et al., 2020). Consequently, we prepared RGD modified SF-based nanoparticles enveloped with Ato to evaluate the hypoxia *in vitro*. Encouraged by the surprising OCR in the RSA-Dox-Ato treatment group, its triggered ROS level was visually monitored by using reactive oxygen species (ROS) analysis kit. ROS production was indicated as green fluorescence (490/525 nm), and the cell nucleus was marked with blue fluorescence (Hoechst 33,342). In addition, the Rosup was an induced agent to activate ROS *in vitro*. As shown in Figure 3C, under a fluorescence microscope, stronger blue and green fluorescence were observed in RSA-Dox-Ato + Rosup group. In contrast, the RSA-DOX + Rosup group had weaker fluorescence. The fluorescence intensity of the RSA-Dox-Ato + Rosup group was higher than that of the other groups, as shown in Figure 3C and Supplementary Figure S4. These results demonstrated that Ato enabled the mitochondrial respiratory to inhibit for alleviating tumor hypoxia, which is consistent with previous investigations (as seen in Figure 1F). Collectively, RSA-Dox-Ato NPs prepared in this experiment could reduce oxygen consumption by inhibiting the function of mitochondrial complex, further improving ROS level for alleviating tumor hypoxia, which is helpful against tumor chemotherapy.

### Antitumor Effect *In Vivo*

**FIGURE 4:**
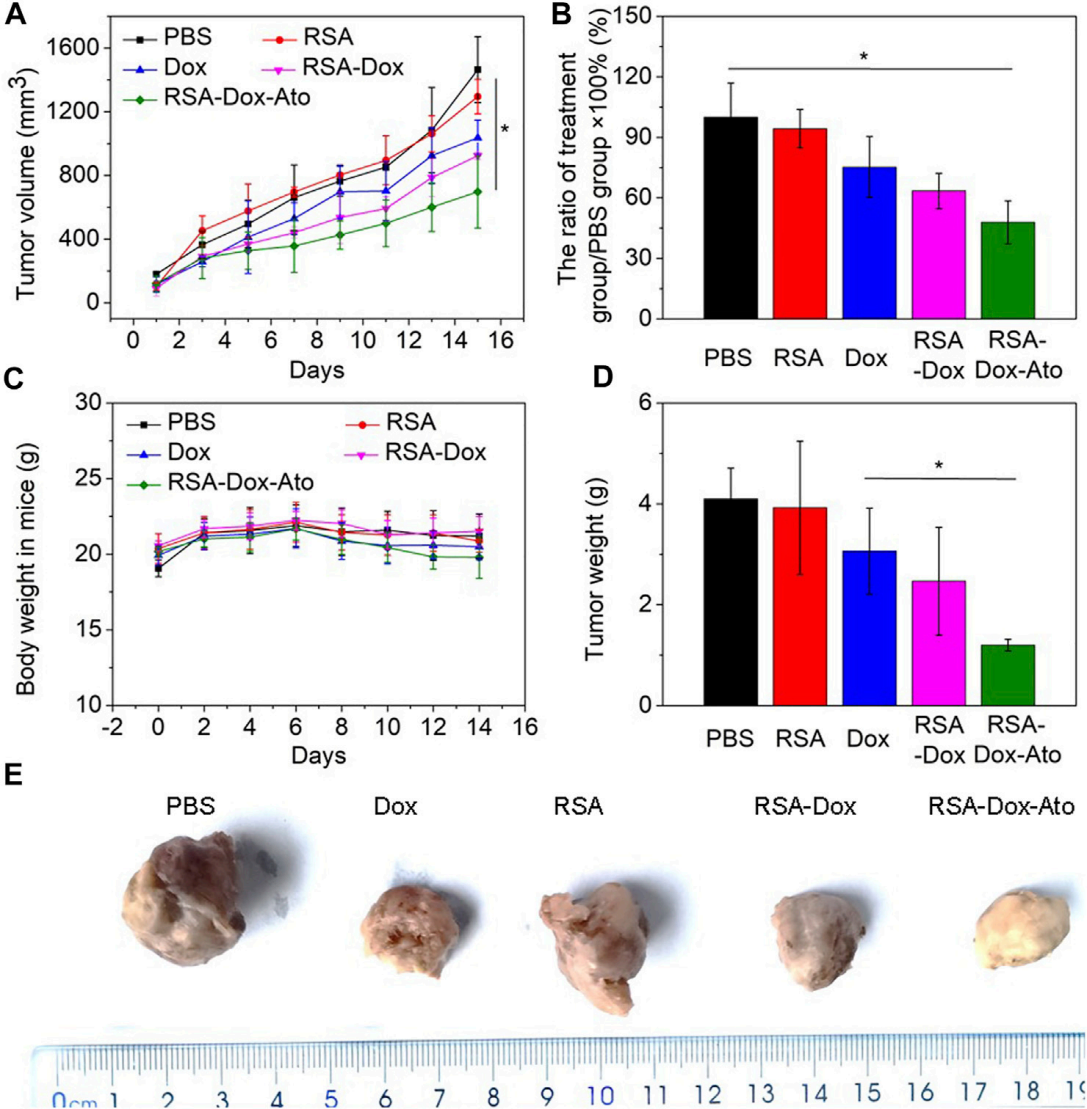
The behavior of RSA-Dox-Ato NPs post-i.v. injection *in vivo*. **(A)** The tumor volume growth curves of 4T1 tumor-bearing mice injected with nanodrugs for 14 days. The data were represented as the mean ± SD (*n* = 5, ∗*p* < 0.05, ∗∗*p* < 0.01); **(B)** The tumor growth inhibition ratio of each group; **(C)** The body weight curve of 4T1 tumor-bearing mice upon the period of treatment; **(D)** The tumor weight curve of 4T1 tumor-bearing mice upon the period of treatment; **(E)** Representative photos of tumor-bearing mice from different treatment groups at the end of the study.

Encouraged by the satisfactory therapeutic effect *in vitro*, RSA nanoparticles had a very high probability of achieving effective cancer treatment for breast cancer. In order to verify the remarkable anti-tumor effect of RSA nanodrugs, further 4T1 tumor-bearing mice were prepared to investigate the behavior of RSA-Dox-Ato *in vivo*. As shown in Figure 4A, the mice were injected with PBS as the control group, and other mice treated intravenously with the Dox, RSA NPs, RSA-Dox NPs, and RSA-Dox-Ato NPs, respectively. The RSA-Dox group exhibited a retarded tumor development within 14 days, compared with PBS and RSA groups. Importantly, it was confirmed by the significant inhibition ratio for the tumor volume in the RSA-Dox-Ato NPs treated mice when compared to the other treatment groups, indicating the RSA-Dox-Ato contributed to retard tumor development as a promising drug delivery system (as seen in Figure 4A and Figure 4B). By monitoring the weight of mice within the treatment period, it was found that the weight change of the mice treated with nanodrugs was unobvious (Figure 4C). Meanwhile, the tumors were weighted for evidencing the inhibition of RSA-Dox-Ato NPs as shown in Figure 4D. Then, the mice were dissected, and the tumors were harvested and fixed in 4% (w/v) paraformaldehyde solution (as displayed in Figure 4E). There was a significant suppression for the tumor weight of treatment groups in comparison with the control group, indicating the excellent antitumor efficacy of the SF-based nanocarriers. As a result, the inhibition tendency of tumor weight was consistent with Figure 4A.

### Alleviation of Tumor Hypoxia and the Inhibition of Lung Metastasis

**FIGURE 5:**
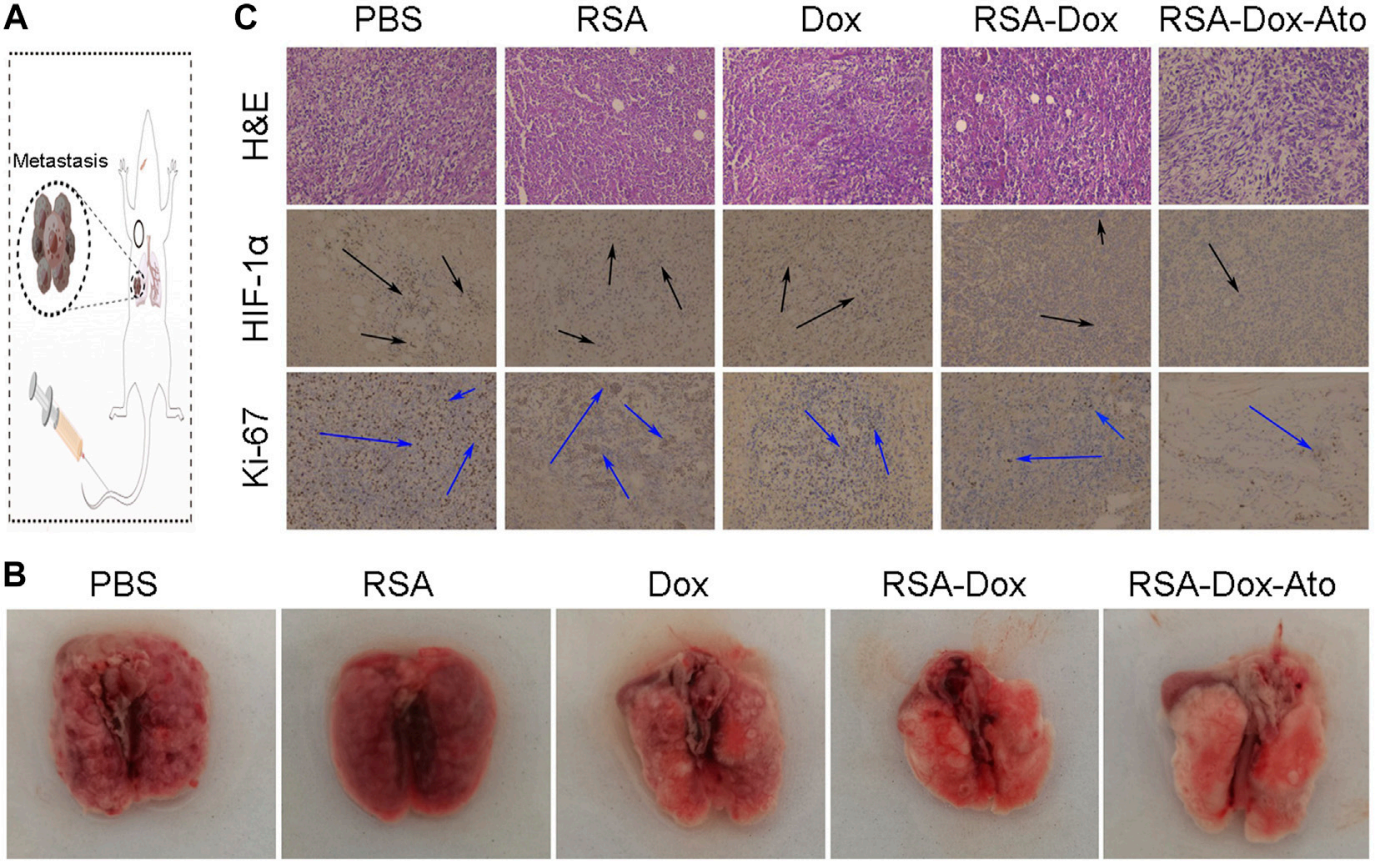
The pathological analysis and lung metastasis of mice received RSA-Dox- Ato NPs with the intravenous injection (*n* = 3). **(A)** Scheme of RSA-Dox-Ato NPs against tumors. **(B)** Representative images of tumor metastasis were collected from mice after treatments with RSA-Dox-Ato NPs. **(C)** The pathological sections were analyzed by unitizing H &E staining, HIF-1α, and Ki-67 (×10 objective).

Dox, as an inducer of immunogenic cell death ICD, activated the immune system by abscopal effect to inhibit tumor metastasis. Enhanced chemotherapy of RSA-Dox-Ato NPs were enable Dox to enhance ICD occurrence *in vivo*, which was beneficial to inhibit tumor metastasis. As a proof-of-concept, the metastasis inhibition of 4T1 tumor-bearing mice was investigated by injected intravenously with RSA-Dox-Ato NPs. As exhibited in Figures 5A,B, there were the most significant lung metastases in the PBS group than the other groups. It was noted that the RSA-Dox-Ato group had the least lung metastasis, indicating that SF-based nanocarriers loaded with Dox and Ato had a better inhibition effect on tumor metastasis. Given the outstanding therapeutic efficacy of RSA-Dox-Ato NPs compared with other treatment mice, tumor hypoxia as an important factor was evaluated *in vivo*. When an imbalance between oxygen supply and consumption, the hypoxia-inducible factor 1α (HIF-1α) was often induced to overexpress in the tumor cell. Based on this, the hypoxia-inducible factor 1α (HIF-1α) in the tumor section was stained with an immunohistochemical method to carried out for evaluation of tumor hypoxia *in vivo*. Compared to other treatment groups, the expression of HIF-1α in the RSA-Dox-Ato group was significantly lower (as exhibited in Figure 5C). Furtherly, all samples from 4T1 tumor-bearing mice after *in vivo* treatment with SF-based nanocarriers were evaluated utilizing pathological analysis. As shown in Figure 5C, the tumor sections stained with hematoxylin and eosin (H&E) from the RSA-Dox-Ato group showed obvious necrotic areas, while no significant damage was observed in the PBS group. From the result of the Ki-67 stained, the RSA-Dox-Ato group revealed the most negligible proliferation factor than that in other groups. Together with H&E staining analysis, the relief of hypoxia (HIF-1α), and low levels of proliferation marker Ki-67, the RSA-Dox-Ato NPs had excellent behavior *in vivo*.

### The Evaluation of Safety *In Vivo*

**FIGURE 6:**
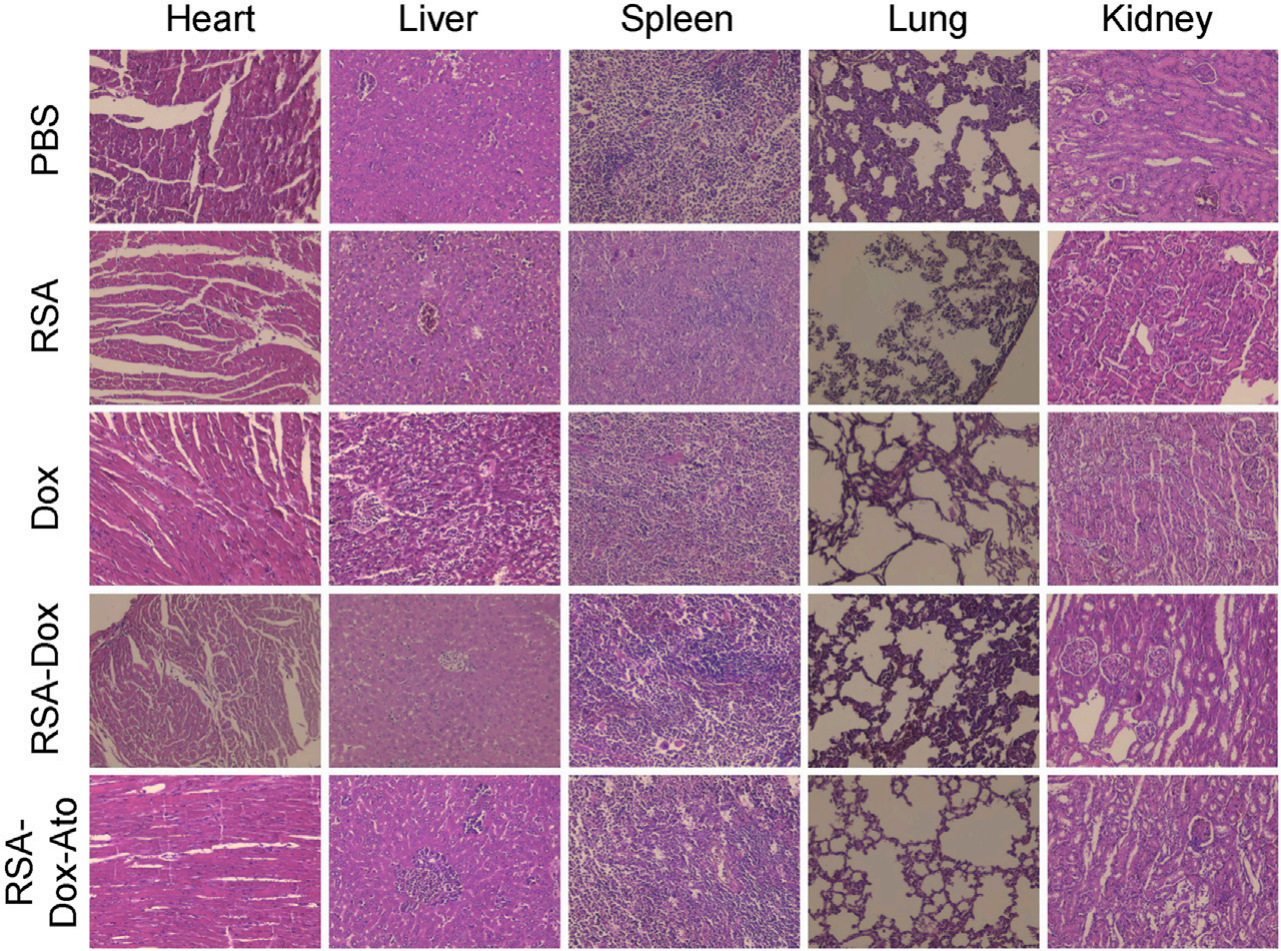
The primary safety was evaluated by H&E staining of major organs from treatment mice (×10 objective).

Silk fibroin, as drug delivery materials, was often applied in the clinic owing to its excellent biocompatibility and biodegradability. Therefore, the primary safety assessment was employed using H&E staining of major organs from mice, as indicated in Figure 6. Compared with the PBS group, there were ignorable changes in heart, liver, spleen, lung, and kidney in the RSA-Dox-Ato group, indicating that the RSA-Dox-Ato had better biocompatibility as a promising value for antitumor application in the future.

## Discussion

In summary, the RGD modified SF-based nanocarriers were successfully custom-designed, which could suppress mitochondrial respiration to alleviate the hypoxia microenvironment. This versatile platform played a significant role in drug delivery and active targeting to improve antitumor efficacy. Significantly, the tumor hypoxia microenvironment had been alleviated by biodegradable SF-based nanomicelles, eventually rendering systematic chemotherapy against tumor metastasis. As expected, RGD modified SF-based nanomicelles as a biodegradable drug delivery platform inhibited primary tumor and metastasis, lighting up an alternative biomaterial in clinical application.

## Data Availability

The original contributions presented in the study are included in the article/Supplementary Material, further inquiries can be directed to the corresponding authors.
