# Supplementary material for: Biodegradable Silk Fibroin Nanocarriers to Modulate Hypoxia Tumor Microenvironment Favoring Enhanced Chemotherapy
Source: Front Bioeng Biotechnol. 2022 Jul 22;10:960501. doi: 10.3389/fbioe.2022.960501 (PMC9354019; doi:10.3389/fbioe.2022.960501)
Supplement: Supplementary file 1 [file Image1.pdf]

Supplementary Material

1.1 Supplementary Figures

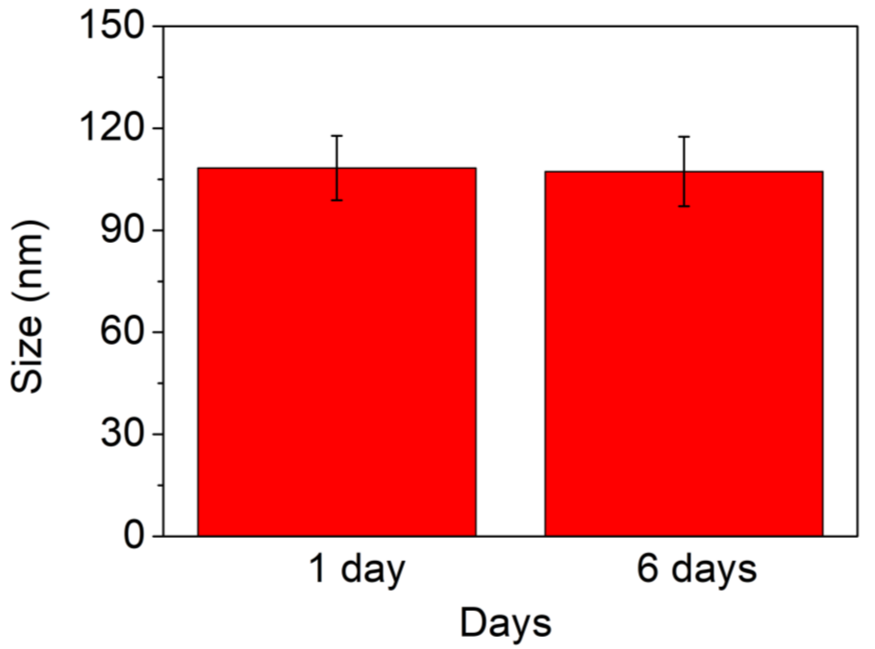

Supplementary Figure 1. The mean hydrodynamic diameter of RSA-Dox-Ato NPs was monitored by DLS under room temperature for 6 days.

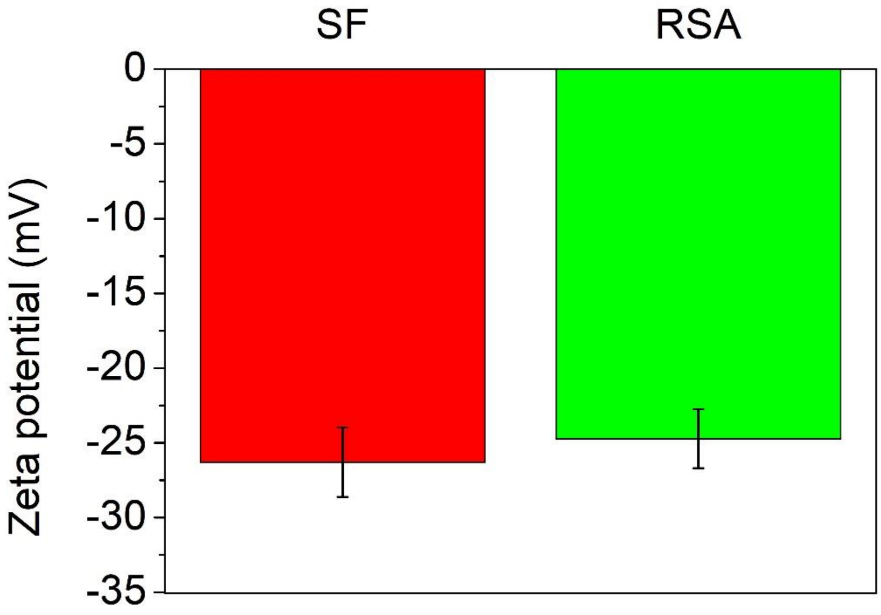

Supplementary Figure 2. The zeta potential of nanoparticles.

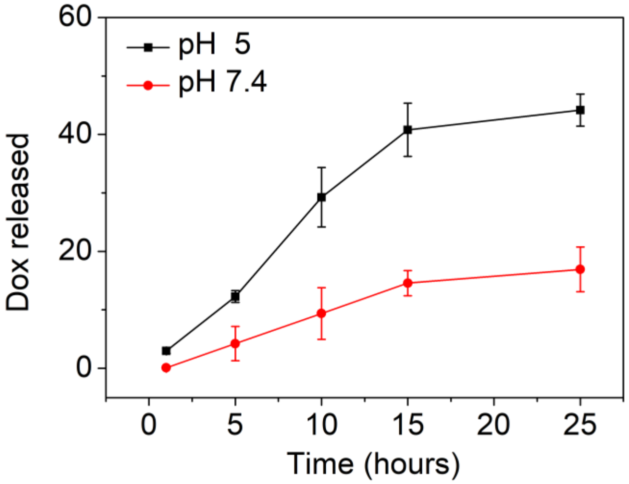

Supplementary Figure 3. The release of RSA-Dox-Ato under different pH condition

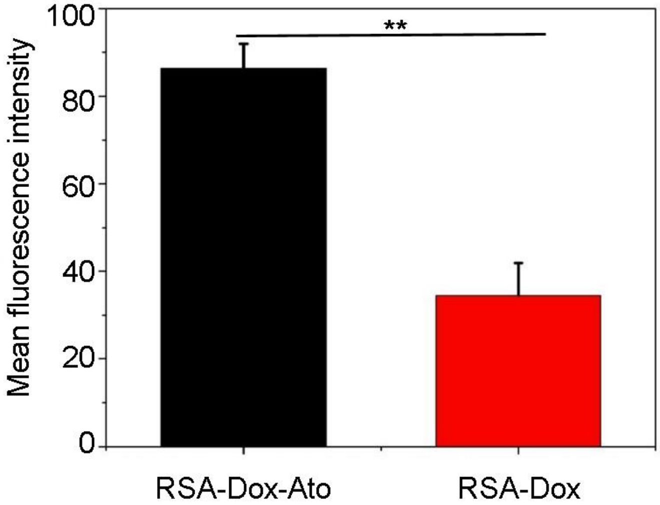

Supplementary Figure 4. The mean fluorescence intensity of RSA-Dox-Ato in 4T1 tumor cells.
